# Isolation of *Mycobacterium avium subspecies paratuberculosis* from Ugandan cattle and strain differentiation using optimised DNA typing techniques

**DOI:** 10.1186/1746-6148-8-99

**Published:** 2012-06-29

**Authors:** Julius Boniface Okuni, Chrysostomos I Dovas, Panayiotis Loukopoulos, Ilias G Bouzalas, David Patrick Kateete, Moses L Joloba, Lonzy Ojok

**Affiliations:** 1 College of Veterinary Medicine, Animal Resources and Biosecurity, Makerere University, P.O.Box 7062, Kampala, Uganda; 2 Faculty of Veterinary Medicine, Aristotle University of Thessaloniki, Thessaloniki, 54124, Greece; 3 Department of Medical Microbiology, College of Health Sciences, Makerere University, P.O.Box 7062, Kampala, Uganda

**Keywords:** *Mycobacterium avium subspecies paratuberculosis*, Cattle, Uganda, SSR, MIRUs, Genotyping, IS*1311* PCR-REA

## Abstract

**Background:**

The occurrence of paratuberculosis in Ugandan cattle has recently been reported but there is no information on the strains of *Mycobacterium avium subspecies paratuberculosis* (MAP) responsible for the disease. The aim of this study was to isolate and characterise MAP from seropositive cattle and paratuberculosis lesions in tissues obtained from slaughtered cattle in Uganda.

**Results:**

Twenty one isolates of MAP were differentiated into 11 genotype profiles using seven genotyping loci consisting of Insertion Sequence 1311(IS*1311*), Mycobacterial interspersed repeat units (MIRU) (loci 2, 3), Variable number tandem repeats (VNTR) locus 32 and Short sequence repeats (SSR) (loci 1, 2 and 8). Three different IS*1311* types and three MIRU 2 profiles (7, 9, 15 repeats) were observed. Two allelic variants were found based on MIRU 3 (1, 5 repeats), while VNTR 32 showed no polymorphism in any of the isolates from which it was successfully amplified. SSR Locus 1 revealed 6 and 7 G1 repeats among the isolates whereas SSR locus 2 revealed 10, 11 and 12 G2 repeats. SSR locus 8 was the most polymorphic locus. Phylogenetic analysis of SSR locus 8 sequences based on their single nucleotide polymorphisms separated the isolates into 8 genotypes. We found that the use of Ethylene glycol as a PCR additive improved the efficiency of the PCR reactions for MIRUs (2, 3), VNTR 32 and SSR (loci 1 and 2).

**Conclusions:**

There is a high strain diversity of MAP in Uganda since 21 isolates could be classified into 11 genotypes. The combination of the seven loci used in this study results into a very precise discrimination of isolates. However analysis of SNPs on locus alone 8 is very close to this combination. Most of the genotypes in this study are novel since they differed in one or more loci from other isolates of cattle origin in different studies. The large number of MAP strains within a relatively small area of the country implies that the epidemiology of paratuberculosis in Uganda may be complicated and needs further investigation. Finally, the use of Ethylene glycol as a PCR additive increases the efficiency of PCR amplification of difficult templates.

## Background

*Mycobacterium avium subspecies paratuberculosis* (MAP) is the causative agent of paratuberculosis or Johne’s disease; a chronic intractable enteritis which affects many species of animals including cattle, goats, sheep and other domestic and wild ruminants [1]. Paratuberculosis poses a serious economic challenge where it occurs, especially in dairy cattle [2]. Some reports indicate that MAP may be involved in the causation of Crohn’s disease (CD), a human chronic enteropathy whose lesions resemble those of paratuberculosis [3]. It has also been reported that MAP from CD patients and animal species have similar genetic patterns [4]. The seroprevalence of paratuberculosis in Ugandan cattle has been estimated to be 8.8% [5]. However, the host range of MAP in Uganda is still unknown and so is the diversity of infecting strains. Furthermore, there are no reports on the diversity of MAP from any African country at the moment.

Molecular characterisation of MAP was initially based on Restriction Fragment Length Polymorphism using hybridization to MAP specific insertion sequence - IS*900* (RFLP-IS*900*); and Pulsed Field Gel Electrophoresis (PFGE) [6,7]. These methods are cumbersome, costly and also require large amounts of purified DNA which can only be obtained from cultures.

The first PCR based characterisation method based on restriction enzyme analysis of insertion sequence 1311 (IS*1311* PCR-REA) and Heat shock protein 65 gene, used limited amounts of DNA but could not distinguish between most isolates [8,9]. IS*1311* PCR-REA is unable to distinguish between most strains except the simple classification of MAP as cattle (C), sheep (S) and bison (B) types [10,11]. Further advances in molecular typing of mycobacteria introduced the use of mycobacterial interspersed repeat units (MIRU) [12,13], variable number tandem repeats (VNTR) [13,14] and short sequence repeats (SSR) [4,15] that have enabled the discrimination between two isolates from the same animal [13]. An additional advantage of these systems is that they require minimal quantity of DNA. Amonsin et al. [16] identified 11 SSR loci, three of which have been used in several studies for genotyping [4,15].

El-Sayed et al. [15] evaluated nine MIRU loci, three SSR loci and six VNTR loci, for characterisation of MAP using 34 isolates of German origin. They recommended the combined use of MIRU locus 2 and SSR loci 1, 2 and 8 (G1, G2 and GGT repeats), although MIRU locus 3 was also discriminatory. They found VNTR loci to be less discriminatory and not very suitable for MAP characterisation. However, other studies that used different sets of VNTR loci indicated that loci 25, 32 and 292 could have some useful discriminatory ability [4,13,14]. There is however conflicting information between Thibault et al. [14] and Castellanos et al. [13] as to whether VNTR 32 or VNTR 25 has a higher Hunter-Gaston index of diversity. Thibault et al. [14] studied 183 isolates from 10 different countries (USA, Venezuela, Argentina, France, Italy, Czech Republic, New Zealand, UK, Slovenia, and Netherlands), while Castellanos et al. [13] used 70 isolates from only Spain. The differences observed might have been due to geographical differences or genetic makeup of the cattle in the different study populations.

In this paper we report the existence of a wide range of MAP genotypes from cattle in Uganda as determined using MIRU-VNTR and SSR typing, in combination with IS*1311* PCR-REA. This is the first report on characterisation of MAP from cattle in Uganda and from any African country. We also present some modifications to improve the current PCR procedures used in the amplification of some loci.

## Results

**Figure 1:**
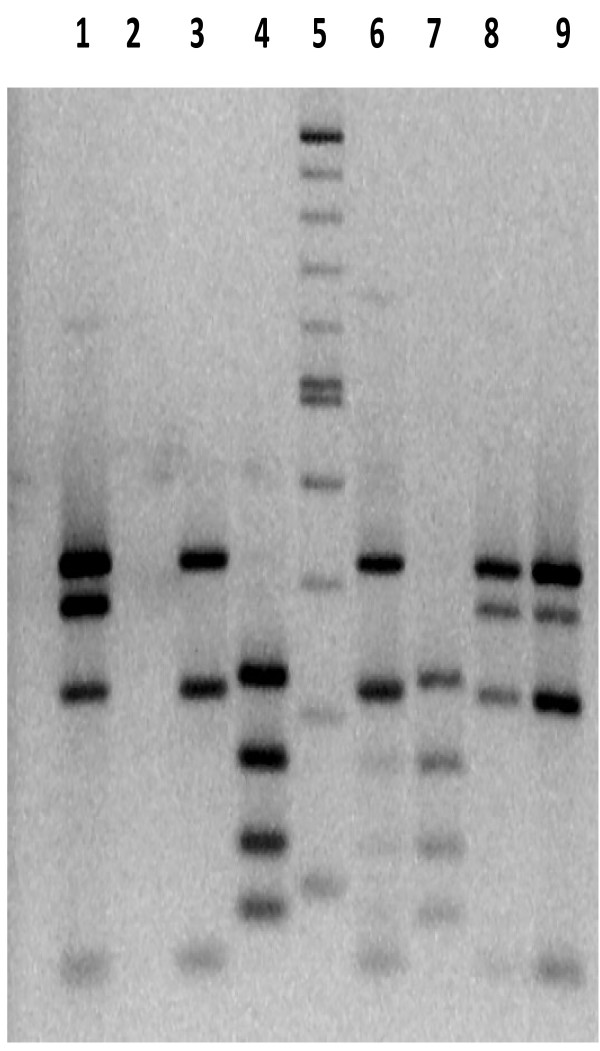
**Restriction fragment length polymorphisms of the IS*1311*PCR product (608-bp), from selected MAP isolates.** Digestion was performed using *HinfI.* Lane 5: 100 bp DNA marker, lane 1: positive control (MAP K10 strain), lane 2: no template control, lanes 3 and 6: bison type isolates MapUg1 and 3 respectively, lanes 8 and 9: cattle type isolates MapUg10 and 17 respectively, lanes 4 and 7: unreported type designated as X and represented by isolates MapUg2 and 4 respectively.

Twenty four isolates were obtained from culture of tissue and faecal materials. Twenty one of them were positive for IS*900* PCR and IS*1311* PCR yielding 229 bp and 608 bp respectively. None of the 21 isolates which were positive for IS*900* was positive for IS*901* PCR ruling out their classification as *M. avium avium and M. avium silvaticum*. Digestion of *IS1311* PCR products with *HinfI* showed that seventeen isolates were of cattle type, two were of the bison type whereas two others had a novel restriction pattern and were classified as belonging to an unknown type designated here as X (Figure 1, Table 1). The cattle type has resulted in fragments of 323, 285, 218, 67 bases while the bison type has resulted in fragments of 323 and 285 bases. On the other hand type X has fragments of approximately 225, 175, 120, 90 base pairs estimated from the ladder.

**Table 1:** Genotypes of MAP isolates in this study and their origins

		**Genotyping method**								
**Isolate No.**	**Genotype No.**	**No. of MIRU repeats (Product size)**			**Short sequence repeats (Number)**			**IS*1311*/REA type**	**Herd code**	**DISTRICT**
		**MIRU 2**	**MIRU 3**	**VNTR 32**	**Locus 1**	**Locus 2**	**Locus 8**			
MapUg1	1	9(350)	1(198)	-	-	12 G	GGTtgt**GGT**	B	020	Wakiso
MapUg2	2	15(520)	1(198)	-	-	11 G	GGTtgt**GGT**	X	058	Luwero
MapUg3	3	-	-	-	-	11 G	GGTtgt**GGT**	B	013	Wakiso
MapUg4	4	9(350)	5(300)	9(300)	-	11 G	GGTtgt**GGT**	X	018	Wakiso
MapUg5	5	9(350)	5(300)	9(300)	-	10 G	GGTcgt**cgt**	C	022	Wakiso
MapUg6_	6	15(520)	5(300)	9(300)	6 G	10 G	GGTtgt**GGT**	C	023	Wakiso
MapUg7	7	7(300)	5(300)	9(300)	7 G	10 G	4GGT	C	030	Wakiso
MapUg8	8	15(520)	5(300)	9(300)	7 G	11 G	GGTtgt**GGT**	C	044	Luwero
MapUg9	8	15(520)	5(300)	9(300)	7 G	11 G	GGTtgt**GGT**	C	038	Mpigi
MapUg10*	9	15(520)	5(300)	9(300)	-	11 G	GGTtgt**GGT**	C	033	Mpigi
MapUg11	10	9(350)	5(300)	9(300)	6 G	10 G	4GGT	C	048	Masindi
MapUg12	10	9(350)	5(300)	9(300)	6 G	10 G	4GGT	C	Abattoir	Kampala
MapUg13	10	9(350)	5(300)	9(300)	6 G	10 G	4GGT	C	Abattoir	Kampala
MapUg14	10	9(350)	5(300)	9(300)	6 G	10 G	4GGT	C	Abattoir	Kampala
MapUg15	10	9(350)	5(300)	9(300)	6 G	10 G	4GGT	C	Abattoir	Kampala
MapUg16	10	9(350)	5(300)	9(300)	6 G	10 G	4GGT	C	Abattoir	Kampala
MapUg17	10	9(350)	5(300)	9(300)	6 G	10 G	4GGT	C	Abattoir	Kampala
MapUg18	10	9(350)	5(300)	9(300)	6 G	10 G	4GGT	C	Abattoir	Kampala
MapUg19	10	9(350)	5(300)	9(300)	6 G	10 G	4GGT	C	Abattoir	Kampala
MapUg20	10	9(350)	5(300)	9(300)	6 G	10 G	4GGT	C	Abattoir	Kampala
MapUg21	11	9(350)	5(300)	9(300)	6 G	10 G	2GGT	C	Abattoir	Kampala

Genotype profiles of the 21 isolates. Note that isolates whose MIRUs, VNTR 32, SSR loci 1, 2 could not be amplified had distinct genotype profiles. These isolates were confirmed to belong to different genotypes using the SSR locus 8 phyologenetic analysis.

* MapUg10 belonged to a different genotype according to SSR locus 8 SNP phylogenetic analysis.

**Figure 2:**
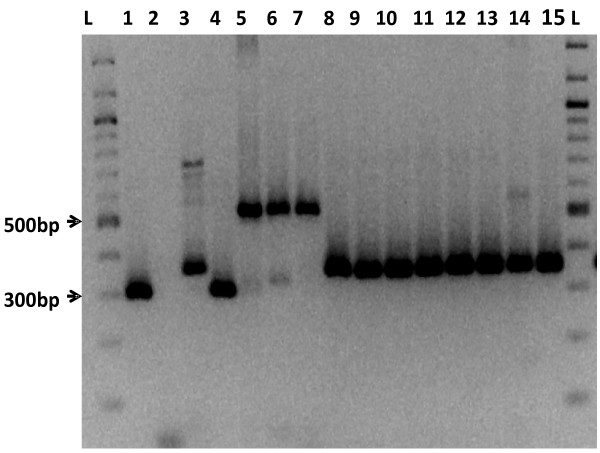
**Agarose gel electrophoretic analysis of MIRU 2 profiles from selected MAP isolates.** L: 100 bp DNA marker, lane 2: no template control, lane 1: MAP reference strain K10 (7 repeats), Lane 4: isolate with 7 repeats, Lanes 3, 8 to 15: isolates with 9 repeats, Lanes 5, 6, 7: isolates with 15 repeats.

Three MIRU 2 allele variants were found among the isolates (Table 1): the first type had seven repeats of approximately 300 bp and was found in one isolate; the second type had nine repeats of approximately 350 bp and was observed in 14 isolates and the third type carried 15 repeats of approximately 520 bp observed in five isolates (Figure 2). Two bands were observed in isolate MapUg3. Therefore, this isolate was considered as one of those isolates in which MIRU 2 amplification was not successful. Based on MIRU 3 profiles, two allele variants were found: one repeat (198 bp) in two isolates and five repeats (300 bp) in 18 isolates. Only 18 isolates were successfully amplified with VNTR 32 and all belonged to the same allele type with nine repeats (300 bp).

**Figure 3:**
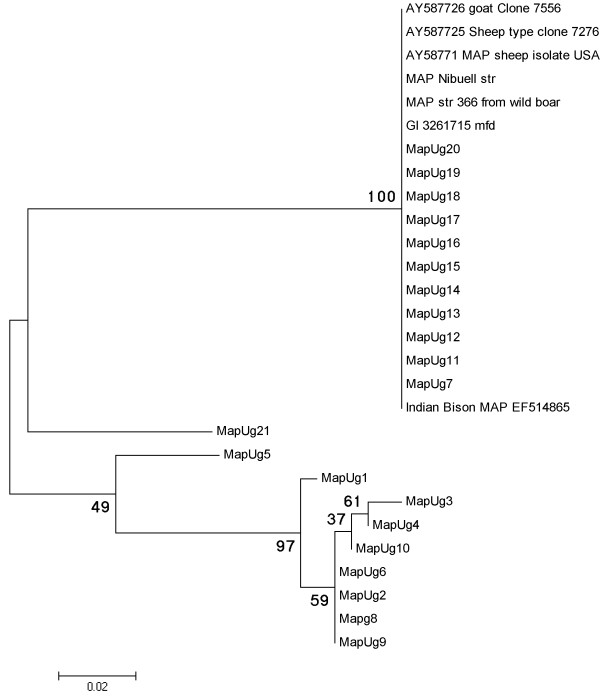
**Phylogenetic tree of homologous partial SSR locus 8 sequences (289 bp) inferred with maximum likelihood analysis based on the Tamura-Nei model.** The numbers on each branch are the non-parametric bootstrap (NPB) probabilities. Sequences represent the 21 isolates (MapUg1 – MapUg21) compared with GenBank records including the reference strain MAP strain K10 and others. All GenBank records including the sheep, cattle and bison type clustered together with 11 isolates whereas the remaining isolates were distinguished into 7 different types.

Regarding the SSR typing, SSR locus 1 amplification was successful in only 15 isolates with a 450 bp product. Sequencing of the PCR products resulted in two allele variants: 12 isolates with six G1 repeats and three isolates with seven G1 repeats. SSR locus 2 was successfully amplified from all the 21 isolates giving a 442 bp product. Sequencing of the PCR products revealed three allele variants: one with 10 G2 repeats in 14 isolates, a second with 11 G2 repeats in six isolates and a third one with 12 G2 repeat in one isolate. SSR locus 8 PCR resulted in approximately 355 bp products. With subsequent sequence analysis, this was the most informative of all loci for genotyping. Three allele profiles were identified bearing trinucleotide repeats: 2GGT in one isolate, 4GGT in 11 isolates and 3GGT with mutations, in nine isolates (GGTtgtGGT and GGTcgtcgt) (Table 1). The phylogenetic analysis of SSR locus 8 partial sequences was even more informative and separated the isolates into 8 genotypes based on their nucleotide polymorphisms (Figure 3). This separation of isolates was in general agreement to that revealed by the combination of MIRU, SSR and IS*1311* PCR-REA typing results. However, the above mentioned combination of loci was able to differentiate into three genotypes the isolates MapUg2, MapUg6, MapUg8 and MapUg9, all of which were grouped together as a single genotype by the phylogenetic analysis of SSR locus 8 (Figure 3). Similarly, MapUg7 was also discriminated from isolates MapUg11-Ug20. As a result the combination of all analyses was more discriminative than the SSR locus 8 SNPs analysis alone.

In general, the combination of IS*1311*, MIRU 2, 3; VNTR 32; and SSR loci 1, 2 and 8, resolved the 21 MAP isolates into 11 distinct genotypes which were distributed in the five districts from which the samples were obtained. The details of the different isolates and their genotype profiles are shown in Table 1.

**Figure 4:**
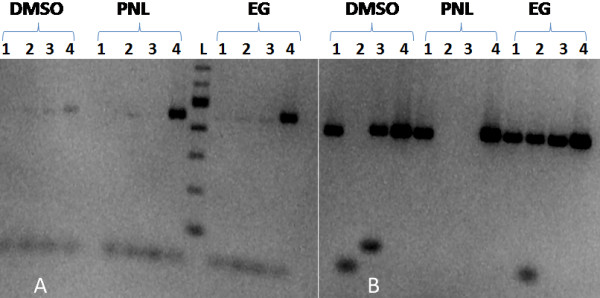
**Agarose gel electrophoretic analysis showing the efficiency of various PCR additives in the amplification of GC rich DNA templates.** The additives were DMSO (7.5%), Propan-1, 2-diol (PNL) (0.816 M), and Ethylene glycol (EG) (1.075 M). Numbers 1, 2, 3 represent three different MAP isolates which were difficult templates to amplify, while 4 represents K10 strain. L: 100 bp DNA marker. On the left (**A**) are PCR products from amplification of SSR locus 1, while on the right (**B**) are products of SSR locus 2. Best results were obtained using Ethylene glycol.

The best amplification reaction for MIRU, VNTR and SSR was obtained using Ethylene glycol at a concentration of 1.075 M (Figure 4). DMSO was the second best denaturant (at a concentration of 7.5%) followed by Propan-1, 2-diol (0.816 M), then Betaine, which was also better than a PCR reaction without any denaturant. The thermal profile was improved by increasing the cyclic denaturation temperature to 97 °C.

## Discussion

This study aimed at determining the strain diversity of MAP isolates responsible for paratuberculosis in Ugandan cattle and comparing them to strains reported from other countries. The results show that most of the MAP strains examined in the study (17 of 21) belonged to the cattle type of strains, while two isolates were of the bison type. A novel restriction pattern was revealed in two isolates (MapUg4 and MapUg2) indicating a type that has not been previously reported. This type was tentatively designated as X pending further characterisation. Cattle strains have been found in most areas where MAP has been detected in cattle. The bison type was first reported in the USA on cattle farms where bison infected with the same strain had been previously kept [10,17,18]. This strain has been reported to be widespread in India among buffaloes, cattle and goats [17,19]. To our knowledge this is the first time the bison type is observed outside the USA and India. Since this is the first characterisation study of MAP in Uganda, it is not clear how widespread this type might be but it has the potential of spreading into more hosts than the cattle type [17,20].

Based on the SSR locus 1, 15 isolates were divided into two allele groups having 6 and 7 G mononucleotide repeats respectively; while three allele profiles were revealed for SSR locus 2 (10 G, 11 G and 12 G) and four allele profiles for SSR locus 8: 4GGT, 2GGT and two polymorphic 3GGT repeats carrying mutations respectively, for all 21 isolates examined. Therefore, based on our results, the examination of the three loci is valuable in discriminating MAP isolates in this region, as has also been shown by El-Sayed et al. [15]. Although it has been reported that loci 1 and 8 were the most discriminative [20], in our study the three SSR loci complement each other and the highest level of discrimination is reached when all the three are used.

Harris et al. [21], found that the majority of the 211 isolates from the USA had more than 14 G1 and 9 G2 repeats for SSR loci 1 and 2 respectively and 5 GGT for SSR locus 8. On the other hand, El-Sayed et al. [15] found 7–14 G1 repeats, the majority being 7 G1 repeats, for SSR locus 1; 9–13 G2 repeats for SSR locus 2, the most common being 11 G among 34 German isolates. Regarding SSR locus 8, El-Sayed et al. [15] found that their German isolates had 4GGT and 5GGT repeats. In another study where SSR has been used, the most common profiles of SSR loci 1, 2 and 8 were >14 G-9 G-5GGT, >14-10-5GGT [21]. No study prior to this one has shown the occurrence of MAP strains with 6 G1 repeats in SSR locus 1, and 2GGT. Polymorphic 3GGT repeats has been observed in sheep strain [4] but the nature of the polymorphism was not defined.

According to Sevilla et al. [20] and Motiwala et al. [22], there is a relationship between the number of repeats in the three SSR loci of a particular strain and the host species. For instance, analysis of SSR locus 2 (G2 repeats) showed that 7 G were found in isolates from bison, impala, nyala, Thomson’s gazelles and goat, while 9 G were found in isolates from duikers, transcaspian urial and waterbucks. Elks were infected with isolates with 7 G and 13 G [19]. Sevilla et al. [20] observed that cattle isolates in Spain had 8, 9 and 11 G repeats in the same SSR locus, and that sheep and goats were mainly infected with isolates having 3GGT repeats on SSR locus 8. In contrast to the findings of El-Sayed et al. [15] who observed that all isolates with seven or greater number of G1 repeats had 5GGT repeats on SSR locus 8, this was not the case in the present study.

The significance of these SSR genotypes with regards to the potential differences in pathogenesis is not yet known but it suffices to say that the 7 G-4GGT profile seen in one isolate, is associated with MAP isolated from Crohn’s disease patients [4] and has also been found to be the most common type among cattle herds in Ohio, USA [23].

According to the present study, a combination of different loci comprising IS*1311*, MIRU (2, 3), VNTR 32 and SSR (loci 1, 2 and 8) discriminated 21 MAP isolates into 11 distinct strains. This shows very high strain diversity in Uganda. It implies that either the Ugandan isolates have evolved over a very long period of time or that they have all come from different regions in the recent past. Further studies to map out the distribution of these isolates and the historical origins of the cattle might clarify this question. It would also be of interest to determine if the different strains found in this study have any association with any particular breed or genotype of cattle and if the strains themselves differ in their pathogenecity. The most common genotype was number 10 with 10 isolates, with a 9-5-9-6 G-10 G-4GGT profile according to MIRU 2 and 3, VNTR 32 and SSR loci 1, 2 and 8 respectively (Table 1). Nine of these isolates came from abattoir specimens. Although we could not get reliable information on the districts of origin of the cattle from which these isolates were derived, we conjecture that they might be from western Uganda, since the same genotype was seen in a cow from Masindi district which is located in Western Uganda and also due to the fact that most of the cattle slaughtered in these two abattoirs come from western Uganda. The remaining genotype profiles had 1–3 isolates each and were evenly distributed in the districts of Wakiso, Mpigi and Luwero, where there are also several breeds of cattle and different husbandry practices.

Castellanos et al. [13] observed that type II isolates of MAP from Spain had five repeats at MIRU 3 locus, while type III had three repeats in contrast to some German type III isolates which had five repeats at MIRU 3 locus. In our study, 18 of the 20 isolates amplified, had five repeats on MIRU 3 locus indicating that they may be related to type II isolates. However, five of our isolates which had five repeats at MIRU 3 locus also had 15 repeats at MIRU 2. The 15 repeats at MIRU 2 locus has been associated with type I [13,24]. As more strains continue to be isolated, the classification of MAP could become more complex than the simple type I, II and III system that was predominant in the last decade or new criteria will have to be devised to classify them into those types for epidemiological reasons. The isolates in this study differ from isolates reported in other studies especially with regard to their SSR profiles and should therefore be considered as novel strains.

Characterisation using SSR locus 8 SNP profile alone was only slightly less discriminatory than the combination of all three systems: MIRU-VNTR/SSR/IS*1311* PCR-REA. Therefore phylogenetic analysis of SSR locus 8 could offer an opportunity for strain discrimination and follow up of the transmission patterns in the country, but it also failed to distinguish between several diverse isolates including the cattle, sheep and bison types found in GenBank (see Figure 3).

In our case MIRU 2 was found to be more discriminative than MIRU 3 and VNTR 32. This is in agreement with the findings of El-Sayed et al. [15] and Castellanos et al. [13]. It was also possible to amplify MIRU 2 from almost all the DNA templates that we had. Another positive aspect of the use of MIRU 2 is that it distinguishes MAP from *M. intracellulare* in which the locus is not amplified [25]. Finally VNTR 32 was the least discriminative and most difficult to amplify.

As stated in the materials and methods, *in-silico* analysis indicated high melting temperatures (>100 °C) for the GC-rich MIRU, VNTR and SSR templates. This was the possible reason for the failure of amplification we encountered in many cases using published primers and protocols [9,12,15,19] (data not shown). In an effort to improve the efficiency of amplification, new specific primers were designed and several additives such as DMSO, Betaine, 1, 2, Propan-diol and Ethylene glycol that facilitate DNA denaturation [12,26], were tested. The new primers were more effective than published primers both with and without the additives. With the new primers, we were able to obtain products from samples which had been too difficult to amplify despite the use of the additives. A cyclic denaturation step of 97 °C for 30 seconds was also adopted. The best results were obtained using Ethylene glycol. Previous studies on MIRUs and SSR have used DMSO and Betaine [12-14] but in our case those two denaturants were not very effective. Unfortunately despite the improvement of the PCR protocols we were not able to obtain amplicons for some of the isolates especially for SSR locus 1 and VNTR 32. Motiwala et al. [9] were also unable to amplify SSR locus 1 from some isolates.

## Conclusions

There is great diversity among MAP isolates responsible for Paratuberculosis in Uganda. The greatest strain differentiation is obtained using a combination of different markers; however phylogenetic analysis of SSR locus 8 offers the single most discriminating tool for the differentiation of MAP isolates in Uganda. VNTR 32 is difficult to amplify and yet it offers no information on the diversity of MAP whereas, SSR locus 1 offers some information but failure of amplification is possible with some isolates. We conclude that the combined use of *IS1311* PCR-REA, MIRU 2, 3, SSR 2, 8 and phylogenetic analysis of SSR locus 8 is more pragmatic in the characterisation of MAP isolates in Uganda but further studies may be required to validate this combination in other areas.

## Methods

### Isolation of MAP and DNA extraction

MAP was isolated from faeces collected from ELISA positive cattle [5], and tissues that had histological lesions of Johne’s disease. The organisms were cultured on Herrold’s egg yolk medium containing nalidixic acid (100 μg/mL), vancomycin (50 μg/mL), amphotericin B (50 μg/mL) (Sigma, St Louis, Mo, USA) and 2 mg/L of mycobactin J (Institut Pourquier, Montpelier, France) as described by Whipple et al. [27]. Culture was performed at 37 °C for 20 weeks. Colonies were analysed for acid fast bacteria using Ziehl-Neelsen staining. MAP isolates had small glistening, translucent to opaque colonies, 2-3 mm in diameter. They were sub-cultured on slants with and without mycobactin J. Mycobactin dependent isolates were selected for further characterisation. Twenty four mycobacterial isolates were obtained following culture of which 21were confirmed to be MAP. DNA was extracted from the bacteria using a method described by Van Soolingen et al. [28]. The sources of the samples and their geographical origins are shown in Table 1.

### Markers used in the study

#### Design of specific oligonucleotide primers for MIRU-VNTR and SSR

After several unsuccessful optimizations with published primers [9,12,15,19] to amplify MIRU-VNTR and SSR loci, we decided to carry out *in-silico* analysis of the template sequences and design new primers. *In-silico* analysis of the templates using Poland^TM^[29,30] showed a high melting temperature (*Tm*) of the templates ranging from 101 to 110 °C. Therefore we modified the primer sets in order to increase their *Tm*, in an attempt to improve the amplification efficiency for the GC rich DNA templates comprising MIRU, VNTR and SSR. The new primers were evaluated using primer design software (oligoAnalyzer 3.1, Integrated DNA technologies, Inc.). Primer IS*1311* M56 [31] was modified to obtain Primer IS*1311*/auF by inserting K to cater for degeneracy observed on the 8^th^ base in some GenBank sequences. To amplify for IS*900* and IS*901* sequences, we used already published primers [25,32]. The list of primers used and their working conditions is shown on Table 2. The primers were synthesized by Metabion International AG (Martinsried, Germany).

**Table 2:** List of primers, their sequences, amplification conditions and locus details

**Primer name**	**Position on MAP K10 genome, acc. no AE016958**	**Sequence**	**Target**	**Thermal conditions (x = No. of cycles)**	**Reference**
IS*900*/150 C	39946-39969	5´-CCGCTAATTGAGAGATGCGATTGG-3’	IS*900*	95 °C-3 min, 45x: 95 °C-30 sec,61 °C-30 sec; 72 °C-20 sec; 72 °C-3 min	[32]
IS*900*/921	40174-40150	5´-AATCAACTCCAGCAGCGCGGCCTCG-3’			
MA901/373up	Not applicable	5’-CGGATTGCTAACCACGTGGTGTGTG-3’	IS*901*	95 °C-3 min, 45x: 95 °C-30 sec; 60 °C-30 sec; 72 °C-40 sec; 72 °C-3 min	[25]
MA901/956do	Not applicable	5’-GTGCTGCGAGTTGCTTGATGAG-3’			
*IS1311*auF (M56)	33911- 33930	5’-GCGTGAGKCTCTGTGGTGAA-3’	IS*1311*	95 °C-3 min, 45x: 95 °C - 30 sec; 59 °C - 30 sec; 72 °C-40 sec; 72 °C-3 min	[11,19] and this study
M119	33323-33340	5’-ATGACGACCGCTTGGGAG-3’			
M2/F	3253559-3253583	5’-CCTGCTCGACGAACACCTCAA-3’	MIRU 2	95 °C-3 min, 47x: 97 °C-30 sec; 60 °C-30 sec; 72 °C-60 sec; 72 °C-3 min	This study
M2/R	3253860-3253834	5’-CGAAGATCCTGGGACTGGACGAGTTGG-3			
M3/F	4441771-4441797	5’-CGGATCGACATTCACCCTGTCCATTCC-3’	MIRU 3	95 °C-3 min, 47x: 97 °C-30 sec; 57 °C-30 sec; 72 °C-60 sec; 72 °C-3 min	This study
M3/R	4442081-4442059	5’-CGAAGCCCTCCTTACGGAGCAGG-3’			
M32/F	115702-1125726	5’-CAGCGCCACAGGGTTTTTGGTGAAG-3’	VNTR 32	95 °C-3 min, 47x: 97 °C-30 sec; 60 °C-30 sec; 72 °C-60 sec; 72 °C-3 min	This study
M32/R	1126006-1125981	5’-GCGGAAATCCAACAGCAAGGACGGAC-3’			
SRL1F	1793439-1793420	5’- GGTGTTCGGCAAAGTCGTTGTGCC-3	SSR 1	95 °C-3 min, 47x: 97 °C-30 sec; 61 °C-30 sec; 72 °C-40 sec; 72 °C-3 min	This study
SRL1R	1792995-1793014	5’-GCGCGTCAGACTGTGCGGTATGG-3’			
SRL2F	2719265-2719287	5’-CGGCTGCACTTGCACGACTCTAGG-3’	SSR 2	95 °C-3 min, 47x: 97 °C-30 sec; 64 °C-60 sec; 72 °C-40 sec; 72 °C-3 min	This study
SRL2R	2718879-2718902	5’-GCGTAGCGTTGTGGGCTTGGAGG-3’			
SLR8F	1027929-1027950	5’-GCGAGATGTCGACCATCCTGAC-3’	SSR 8	95 °C-3 min, 47x: 97 °C-30 sec; 59 °C-30 sec; 72 °C-20 sec; 72 °C-3 min	This study
SLR8R	1028263-1028283	5’-CGCTCGACGATCAGCGTGTTG-3’			

#### IS900 PCR

Amplification of the MAP specific sequence, IS*900* was performed as described by Vary et al. [32] using primers IS*900*/150 C and IS*900*/921. The reaction mixture contained 1 unit of Platinum® Taq DNA polymerase (Invitrogen Life technologies Ltd), 1× PCR buffer, 200 μM each dATP, dCTP, dGTP and dTTP, 2.5 mM MgCl_2_, 0.2 μM of each specific primer, 1 μl of MAP genomic DNA extract and nuclease free water up to 20 μl.

#### IS901 PCR

All DNA samples were subjected to a PCR reaction to amplify IS*901*, an insertion sequence found in *M. avium subspecies avium* and *M. silvaticum*. IS*901* was amplified with primers: MA901/373up and MA901/956do. The reaction mixture consisted of 2 units of Platinum® Taq DNA polymerase (Invitrogen Life technologies Ltd), 1× PCR buffer, 200 μM each dATP, dCTP, dGTP and dTTP, 1.5 mM MgCl_2_, 0.2 μM of each specific primer, 3 μl of MAP genomic DNA extract and nuclease free water up to 40 μl.

#### IS1311 PCR and restriction analysis

To determine if MAP isolates belonged to the cattle, sheep or bison types, IS*1311* was amplified from IS*900* PCR-positive and IS*901* PCR-negative samples and subjected to restriction analysis. Briefly, a pair of primers, IS*1311*auF-(M56) and M119 [31] were used to amplify the sequence. The reaction mixture consisted of 1 unit of Platinum® *Taq* DNA polymerase (Invitrogen Life technologies Ltd), 1× PCR buffer, 200 μM each dATP, dCTP, dGTP and dTTP, 1.5 mM MgCl_2_, 0.2 μM of each specific primer, 3 μl of MAP genomic DNA extract and nuclease free water up to 20 μl. The PCR products were subjected to restriction analysis using *HinfI* restriction endonuclease (New England Biolabs, Massachusetts, USA) at 37 °C followed by electrophoresis in a 2.5% agarose gel.

#### MIRU-VNTR AND SSR PCR

Several additives were tested as denaturation reagents in the PCR protocols for MIRUs, VNTR and SSRs in an attempt to improve the amplification efficiency for the GC rich templates. These included Betaine (0.8 M), Propan-1, 2-diol (0.816 M), DMSO (5 – 10%), and Ethylene glycol (0.775 – 1.375 M). The final optimised reaction mixture for MIRU loci 2 and 3, VNTR 32 and SSR loci 1, 2 and 8 consisted of 1.5 units of Paq5000^TM^ DNA polymerase (Stratagene, La Jolla, CA, USA), 1× PCR buffer containing 2 mM MgCl_2_, 250 μM each dATP, dCTP, dGTP and dTTP, 0.2 μM of each specific primer, 1 μl of MAP genomic DNA extract, 1.2 μl of Ethylene glycol (1.075 M) and nuclease free water up to 20 μl. The primer pairs used for each specific template along with the thermocycling conditions is shown on Table 2. The number of repeats for each MIRU locus was calculated by matching the size of the PCR products to the hypothetical allele calling table drawn by Castellanos et al. [13].

#### Sequencing and analysis of SSR amplicons

Amplicons from each SSR loci were gel purified using Nucleospin Gel extraction kit II (Macherey-Nagel, Germany) and sequenced using the standard big dye terminator chemistry on an ABI 3100 apparatus (Applied Biosystems). The sequencing primers were SRL1F, for SSR locus 1(G1); SRL2doseq (5’GCCACAACGAAATTCGCCTCAG-3’) for SSR locus 2 (G2) and SRL8R for locus 8 (GGT).

Analysis of the sequences was done using MEGA version 5 [30]. The chromatograms were opened, read and double checked for accuracy of the nucleotides in a trace explorer. The repeat regions were searched using the flanking sequences to ensure that the correct motif was identified. The number of nucleotide repeats were counted and recorded for each sequence. The sequences were then exported to the alignment explorer and aligned pairwise using Clustal W. The alignments were visually checked and manually corrected. The motifs were again searched and the number of repeats verified. For SSR locus 8, a consensus phylogenetic tree was generated for SNPs using maximum likelihood method based on the Tamura-Nei model with 1000 bootstrap replications [33].

#### Ethical consideration

Permission to undertake this study and ethical clearance was granted by the Uganda National Council for science and technology (UNCST) under reference number HS311. Before collecting blood samples from cattle, farm owners were issued with letters to request if blood and faecal samples from their cattle may be collected for the study according to the study protocols. Samples were collected only if they agreed to the procedures.

## Abbreviations

MAP, Mycobacterium avium subspecies paratuberculosis; CD, Crohn’s disease; PCR, Polymerase chain reaction; MIRU, Mycobacterial interspersed repetitive unit; SSR, Short sequence repeats; VNTR, Variable number tandem repeats; IS1311 PCR-REA, A restriction digestion performed on PCR products from amplification of the insertion sequence 1311; SNP, Single nucleotide polymorphism.

## Competing interests

The authors declare that they have no competing interests.

## Authors’ contributions

JBO, LO, CID, PL conceived the study; OJB, CID, PL, LO, MLJ designed the study; JBO, IB, DPK, CID did the laboratory work. CID, IB, JBO analysed the data. JBO, DPK, PL, CID, IB, LO wrote the manuscript; all read and approved the manuscript.
